# Investigating the experiences of family caregivers who shackle people with mental disorders

**DOI:** 10.3389/fpsyt.2023.1062100

**Published:** 2023-07-24

**Authors:** Ira Kusumawaty, Yunike Yunike

**Affiliations:** ^1^Department of Mental Health Nursing, Poltekkes Kemenkes Palembang, Palembang, Indonesia; ^2^Department of Pediatric Nursing, Poltekkes Kemenkes Palembang, Palembang, Indonesia

**Keywords:** family burden, family caregivers, phenomenology, human rights, mental disorder, restraint, shackle, stigma

## Abstract

The mental health literacy gap has resulted in the shackling of people with mental illness by family caregivers. Although shackling violates human rights and impacts physical and psychological health, it still occurs in some countries, such as Indonesia. An in-depth study using the family function approach is needed to distinguish the components behind the application of shackling by families to find solutions to prevent shackling. Thus, this study aims to identify family functions in people with mental disorder (PWMD) care and to create a family care model for PWMD in accordance with the family function approach and recommendations for preventing shackling. This qualitative research used a phenomenological approach, involving eight participants who are family caregivers and live with their patients. Triangulation was conducted by applying interviews with four health cadres to confirm previous information. The process of in-depth interviews and observational data collection methods was carried out until reaching data saturation. The data analysis process used Collaizi's pattern to formulate three main themes, namely confinement as the final solution for the family, the specifics of confinement, and the family's purpose for confinement. The conclusion is that shackling occurs due to a lack of understanding of the impact of shackling and the various limitations experienced, so shackling becomes the last resort when dealing with patients. Peer support is very important for families to prevent the confinement of mentally ill family members. Technological advances are an inherent need in everyday life and must support family caregivers with mental disorders.

## Introduction

The increase in cases of people with mental illness has spiked dramatically, although this has not correlated with an increase in understanding. Only a small portion of the already sizable health budget is dedicated to mental health; existing facilities are insufficient to meet current needs (1). In Indonesia, shackling is known as pasung and, although various policies prohibiting shackling have been launched, in reality, shackling is still used as a solution by the community. According to the Basic Health Research (Riskesdas), at the national level, there was a slight decrease in the percentage of shackling from 14.3% (2013) to 14% (2018) (2). Shackling restricts the physical movement of patients, which has a psychological and physical impact. Trauma, resentment toward family, feeling abandoned, low self-esteem, despair, depression, and suicide are some of the psychological impacts of shackling (3, 4), and physical impairment in the form of muscle atrophy in the legs, hands, and joint contractures can also occur (4, 5). Patients with mental disorders should not be shackled; families should strive to get patients comprehensive therapy (6–8).

The interpersonal framework seen within a family is established after a long journey of multiple personal involvements, characterized by a deep search for harmony between different personalities and striving toward stability and enabling improvement, known as family dynamics (9, 10). The family is not only a basic social unit, but also a very important place for a person's physical and psychological development (11, 12). Each element of the household contributes to influencing the achievement of optimal family functioning. At the same time, this has an impact on the growth of each member and is essential for the orderly operation of the social system. McMaster's process-oriented family functioning theory (13–17), and Skinner's process-oriented family functioning theory (18–20) state that the physical and psychological health of the family is influenced by the process of recognition of various tasks in the family, rather than the family structure. The smoother the process of family features, the better the mental health of the family members.

The family system encourages family members to grow together by implementing a series of family responsibilities, including caring for a person with mental illness, which requires family dynamics to find solutions (21, 22). Lack of understanding and desperation to solve chronic and complex problems result in families preferring to shackle people with mental disorder (PWMD) as a final solution (5, 23). Reasons for shackling include concerns that PWMD will harm others (22, 24), family financial limitations to treat PWMD in hospital (25, 26), myths circulating in the community, and the stigma that having a family member with mental illness is a disgrace (27–30). According to McMaster and Skinner, the family function approach in caring for PWMD can be foundational in directing the family in carrying out its functions. However, in-depth studies on family functions in caring for PWMD according to McMaster and Skinner's theories are still limited; therefore, this study aims to identify family functions in caring for PWMD and create a family care model for PWMD in accordance with the family function approach, as well as presenting recommendations for preventing shackling.

This research is significant in shedding light on the experiences of family caregivers who confine individuals with mental illness. By exploring this topic, this research aims to provide insight into the decision-making process, the challenges faced, and the potential impact on both the caregiver and the shackled individual.

Qualitative research in this area is particularly important as it allows for in-depth exploration of the subjective experiences, perspectives, and emotions of family caregivers. It provides a platform to understand the complex factors that influence the decision to confine, such as lack of understanding, societal stigma, and caregiving burden. In addition, qualitative research allows exploration of the potential psychological, emotional, and social consequences experienced by caregivers and individuals in pasung.

## Method

### Design

This qualitative research utilized a phenomenological approach to gain an in-depth understanding of the experiences of family caregivers in confining individuals with mental disabilities. The research aimed to explore the decision-making process, feelings, consequences, and other related aspects of confinement.

### Participants

Eight participants were selected using purposive sampling techniques. The inclusion criteria were caregivers of individuals with mental disabilities who were living in the same house as the confined individual. The participants were identified through collaboration with health workers at a health service center that provides routine services to families of people with disabilities who have experienced confinement.

### Data collection

In-depth interviews were conducted at the participants' homes, and the researchers observed the participants' facial expressions and gestures during the interviews. Interview guides, voice recorders, and field notes were used as tools for data collection. The interview questions were developed based on the McMaster and Skinner model of family function theory, and the guide was pilot tested and refined before being used in the study. The following is the in-depth interview guide used in this research

1. How does the family feel about this shackles/restraint problem?
2. How does the family environment respond to mental disorders and shackling?
3. What is the decision-making process in the family?
4. Why did the family decide to put the patient in shackles?
5. How do you feel when you see that person in shackles?
6. What is the form of attention between family members?
7. How was the shackle decision made?
8. How long is the detention duration?
9. What are the consequences of shackles?

### Data analysis

The data analysis process involved multiple stages. The researcher read the collected data repeatedly to identify overlapping or repetitive information. Similar or compatible data were then classified and labeled. Themes were formulated by combining and integrating different themes based on the classification. A framework was established to capture the essence of the data. Two raters were involved in the data coding process, and disagreements were resolved through reviewing the codebook and refining the code definitions.

### Trust and ethical considerations

The study prioritized the rights and wellbeing of the participants. Informed consent was obtained from each participant, and their confidentiality, physical and psychological comfort, and equal treatment were ensured. Triangulation was achieved by including four health cadres in the study. Member checking was performed to validate the data gathered during the interviews. The research was conducted within the ethical guidelines of and with approval from the Health Research Ethics Committee of the Palembang Health Polytechnic.

This research was conducted over a period of four months, from November 2021 to March 2022, with an approved ethics certificate obtained in September 2021.

## Results

The process of deliberate sampling, also known as purposive sampling, was used to select participants for this research. The researcher collaborated with health workers at a health service center that provided routine services to families of individuals with disabilities who had experienced confinement. The health workers provided information about families who met the inclusion criteria, which included being caregivers of individuals with mental disabilities who lived in the same house as the confined individual.

The researcher, along with the health workers, approached these families and invited them to participate in the study. The decision to include a family in the research was based on their willingness to participate and if they met the specific criteria. The aim was to select participants who could provide valuable insights into the experiences of family caregivers in confining individuals with mental disabilities.

The deliberate sampling technique allowed the researcher to purposefully select participants who fit the criteria and were likely to provide rich and diverse perspectives on the topic of interest. This approach ensured that the research captured a range of experiences and enabled the exploration of various aspects related to confinement and its impact on families.

### The socio-demographic of participants

Referring to Table 1 in this study, participants were family members who acted as caregivers for people with psychiatric disabilities, consisting of fathers, mothers, brothers, or sisters of respondents. The youngest age was 29 years old, and the oldest age was 47 years old, the majority had a high school education, and resided in Palembang city. The number of participants was determined after knowing that the data collected had reached a saturation point, namely after interviewing eight participants. The information explored included the family's reasons for confinement and the determinants of confinement, the family's understanding in considering confinement, and the form and duration of confinement. This information can describe the family's role as a support system for the patient, which is then translated into thematic statements based on transcripts, categorization, or labeling. The themes were summarized and grouped into elements of imprisonment. The following are the results of article themes from in-depth interviews with caregivers, triangulated based on information from health cadres.

**Table 1:** Demographic participants.

**Code**	**Age**	**Gender**	**Education**	**Relationship**	**Age of**
			Level	With PWMD	PWMD
Caregiver A	40	Female	High school	Mother	20
Caregiver B	41	Female	College	Sibling	35
Caregiver C	29	Female	High school	Daughter	49
Caregiver D	35	Male	High school	Son	52
Caregiver E	40	Male	High school	Sibling	35
Caregiver F	45	Female	High school	Sibling	33
Caregiver G	47	Male	College	Sibling	41
Caregiver H	36	Female	High school	Sibling	30

The following themes are formed based on the categorization of participant information and information on health cadres as a triangulation of sources in this qualitative study.

### Sanctuary as the ultimate solution for the family

#### Lack of understanding and ability of the family to care for the patient

Participant H said that they no longer knew how to care for the patient. Confinement serves a moral purpose as they do not become victims of violence by using restraints due to exposure to harmful acts. As a family, they do not know what to do other than using shackling because they have tried many other methods but without good results. Participant B said that trance is only a cause of mental illness. It is the empty mind that possesses and disturbs the psyche.

The health cadre conveyed information supporting the family's lack of understanding in caring for the patient based on complaints submitted by caregivers. They said they did not know how to take care of the patient; rather than making many people become victims, we should just lock them up.

### Limited understanding of the family about the impact of shackles and weakening of physical health conditions

“Her physical condition is now getting weaker, and she can only lie down. There are no activities done while shackled, I don't know why it's like that.” (Caregiver A) Another participant said that “...even my sister can't walk anymore because she has been lying down for too long. Her legs are getting smaller, and she looks thinner, it's very sad.” “At the beginning of being shackled, his condition worsened, and he often had tantrums, but now he is not angry anymore, and his body is getting thinner, always lying down and weak.“ (F's caregiver).

Health cadres reported similar information that the physical condition of the dispossessed victims was worsening. The condition of the legs, which get smaller as a result of never being used to walk, is common. In addition, there was a strong foul odor coming from the patient's body because the family forced the patient to eat and defecate in the same place.

### Torn psychological state

Caregiver C said that her sick father looked shy when he met other people, his head always looked down when he saw other people coming, and it seemed that his father was ashamed of his condition. Meanwhile, caregiver B said that her brother often talked to himself, sometimes hitting the wall, iron, and wood. Caregiver H revealed her brother's expressions such as crying alone, not responding, and throwing objects around him that are currently shackled.

The caregiver said that PWMD was seen smiling and talking to himself and sometimes crying during home visits. In other places, PWMD would bow his head when dealing with cadres. The family also said that when dealing with a stranger, PWMD would hide his face and not respond when asked to answer questions.

### The heavy burden of being in charge of the family

Participant A said that it is the head of the family who decided to put the patient in pasung or who is responsible for providing for the family. As the participant said, “In my family, my father decides that my younger brother should be shackled because my father decides everything in the family.” Participant D said differently because his father was detected to have a mental illness, so his oldest brother was responsible for providing for the family. Other family members follow his decisions. This is in contrast to participant C, “In our family, the mother decides whether the older sister is shackled or not because the mother works while the father cannot work due to illness. Mom is the decision maker in our family.”

The health cadre recounted the story of one of the caregivers when the decision to confine was first made, usually by those responsible for providing for the family. Other family members may provide input, but the final decision remains with the caregiver.

### The specifications of shackles

#### The form of shackles

Using chains as a means of restraint is common. Locked chains made of iron tied to PWMD's legs and attached to a cupboard in the house are a common sight for patients. As a caregiver, G said that his father shackled his younger brother using iron chains so that his brother would not leave the house and so that he would not worry about the surrounding environment. Participant F said that her sister was shackled using wood and had to be at the back of the house so that there would be no commotion.

Health cadres stated that shackles are generally carried out at home and using iron chains. There is even a shackle room in the house so that the family can still monitor the patient's condition.

#### The condition of the patient determines the length of confinement

Based on information from in-depth interviews, it was found that the deprivation process was carried out for various lengths, ranging from three months to four years. Generally, the confinement is done inside the house using chains. Persons with disabilities remain tied up outside the house, so that confinement behind the house does not interfere with family activities in the house.

Health cadres reported that the duration of confinement varies as it depends on the patient's condition and the decision to confine, as well as the family's readiness to untie.

#### Determination of the location of the containment

“We only set up chains at home so that we can constantly monitor the condition of PWMD,” said caregiver A. Caregiver C reported that her family set up chains in the living room to tie her legs. She can still watch television when her legs are tied. My father designed a special room with an iron door at home so as not to disturb other family members; he is separated from others, afraid of endangering others, caregiver E said.

Health cadres share their experiences when visiting patients' homes. People with disabilities are commonly confined to their homes by being chained or having a special room made for them.

The purpose of the family to carry out restraints.

The development of this theme is based on previously formulated categories, including anxiety about the comfort of a disturbed environment, avoiding getting lost, lack of family understanding, poverty, and stigma.

### Anxiety and environmental comfort

Participant D explained that they applied pasung because they were “worried that my father would disturb the peace of the neighborhood.” Participant G supported this statement: “When he works, he often feels hot, often makes trouble with his friends, and causes mental disorders. He often feels overheated, often makes trouble with his friends, and causes mental disorders. We were forced to put him in jail because he often goes out into the street and chases people around, even at night, he often goes berserk and disturbs public safety and order.” Another participant added that

“during the day and night, he has to be locked up because if he is released, it is very feared that he will disturb the peace of the community.” (Caregiver A)

The researcher confirmed with health cadres about the participants' explanations. They expressed the family's concern that the patient would later commit destructive or even violent acts if the disease recurred. This action would certainly damage the good name of the family. Another cadre expressed the caregiver's concern that her sick father would disturb or hit people around him if he suddenly became angry.

### Avoidance of sufferers from getting lost

“We locked my father up so he couldn't leave the house. We were worried that he wouldn't be able to return home. There was once an experience when my father couldn't return home, and it turned out that he was already in a shop near the house and didn't know the way home. From then on, we agreed to put my father in stocks so that he could stay at home” (Caregiver D). “We put him in a cage to keep him at home, not to go anywhere, because we couldn't watch him, and we were worried that he couldn't come back when he left the house. We love my brother, so we keep him at home” (Caregiver B).

The health cadre conveyed the family's complaint that the confinement was done by the family to prevent PWMD from leaving the house and getting lost. Care is needed to keep PWMD at home.

### Ensure monitoring of the patient's condition

“It's easier for us as a family to keep an eye on our sick sister if she has chains on her legs, so we don't have to worry about doing other things at home,” said participant B. Similarly, participants F and G said that there is no other reason for them to confine their sister, just so that she can be monitored, seen, and can still be well cared for. “Although it is also sad to see her shackled like that.”

The health cadre said that the caregivers who put her in shackles were doing so to make it easier to monitor sick family members, so that they would be less worried about her activities at home. “In the past, we tried to put chains on him, but when we were caught off guard, he tried to leave the house.”

### Poverty that ensnares

“We only work as laborers, pedicab drivers, so we don't have the money to take our sick brother to the hospital. It's difficult for us to eat every day,” said caregiver F. Caregiver E corroborated this and said, “Where? Maybe we can take him to the hospital, but it's still difficult for us to eat every day. If we want to eat, we leave everything to God. We work as onion peelers or sometimes clean fish to sell at the market. Instead of spending money on hospitalization, it's better to use it to buy daily necessities.”

The health cadre also mentioned the economic difficulties of the caregivers. The cadre said that, on average, caregivers of PWMD in pasung are economically disadvantaged families; most, although not all, are poor. Economic limitations are the main reason for not bringing patients to mental hospitals.

### Stigma shackles

One of the caregivers gave a sad statement about stigma, namely, “We come from a rich family, and when my child gets lost and wanders the streets without proper clothes, without bathing, that's why we are ashamed. It makes me sad to have to tie him up. No mother would want to chain her child.” Caregiver C shared the same complaint: “My family believes that his mental health condition will stigmatize the whole family. I want to help him but I can't. It's very heartbreaking.” Caregiver A said, “After all, this illness is a curse due to a lack of faith in God and begins with possession. It hurts so much when people talk about the local language, less than half an ounce, less than a canting, it breaks my heart to hear them making fun of my child.”

PWMD's caregiver suffered from the stigma that befalls health cadres based on the complaint of the caregiver who cried and told of being mocked by neighbors or school children when she saw her child walking in front of the school, which is close to the puskesmas, and was mocked with the term “less secanting”, which means less able to think, and called “crazy...crazy...crazy....”.

Another caregiver of PWMD expressed the same condition when PWMD's family said that their neighbors no longer invite them to gather or talk like they used to when their child was not diagnosed with a mental illness.

## Discussion

Based on the results of the research, there are three main themes, namely confinement as a final solution for the family, the specifications of confinement, and the family's purpose for confinement. Confinement is used as a final solution when dealing with family members who experience mental disorders. Some of the themes formulated included lack of understanding in caring for the patient and the impact of confinement, the heavy burden of being responsible for the family, the final choice of form of confinement, and the consideration of the length of confinement. Furthermore, the reasons for family confinement were concerns about disturbing those around them, avoiding the patient getting lost, optimizing supervision conditions, poverty, and the stigma of mental illness.

The decision to confine a family member with mental illness is the implementation of family functions according to McMaster (14, 31, 32) and Skinner et al. (19, 20). Families are responsible for improving the physical and psychological health of their members. However, in this study, the decision made by the family was not the right decision. This error occurred due to the family's lack of understanding regarding the care of people with disabilities and the impact of confinement.

Caregiver burden is usually the result of providing care to chronically ill patients. The severity of family burden when caring for family members with chronic illnesses has been explored in various studies. The difficulties experienced for years and even decades when caring for family members suffering from mental disorders cause family burden (33–36). This condition has implications for the non-optimal realization of family functions, as proposed by McMaster and Skinner. According to McMaster and Skinner's task attainment, the decision to confine a patient is related to problem solving. Decision-making, which is also the achievement of the family's task to confine the patient, is a tough decision because it has a comprehensive impact on the patient's physical and psychological health. The results revealed that there was an emotional response in the form of compulsion in making the decision to confine the patient. Family values played a role in making the shackling decision. Thus, the family functions involved in making the decision to confine the patient include emotional response, expression, agreement, and rules. According to McMaster and Skinner, internalized emotional states are then manifested in the form of real behavior and appear in family behavior patterns. The fear that PWMD will disturb the environment around the house and even get lost if they are not there, and the desire to be able to supervise PWMD, are the reasons for the emergence of certain behaviors in the form of confinement. Behavior and emotional reactions correlate with the underlying understanding of one's thinking. In this study, inappropriate responses to family functioning and treatment of people with mental disabilities were caused by a lack of knowledge about the impact of confinement in stocks. Although the decision to confine a person with a mental disability is based on mutual agreement among family members, the accuracy of the decision is related to the understanding of all family members. Collaboration and interaction through communication can be positive, but if the family's knowledge is not correct, it can have a negative impact on the patient's condition.

Participants think that by confining the patient, there will be no victims due to PWMD's violent behavior, and caregivers will find it easier to supervise the patient.

Some families do not have the heart to shackle patients, as shown in previous research (8). Affection as a form of family affective function still exists. However, a sense of fatigue and shame because of the patient is considered a family disgrace and curse, causing the family to feel confident to carry out confinement. The family's financial factor also shapes and determines the family's decision to confine the patient. The poverty that ensnares families makes them display the behavior pattern of shackling as a final solution because they feel they cannot take other solutions. The stigma that ensnares some families further motivates them to confine the patient as a form of family function in the form of behavior.

Psychoeducation is important for families to improve family coping. For example, families must care for family members who suffer from mental disorders. Michael Foucault (37–39), a French philosopher, stated that the family is a place to build the bodies of its family members. Thus, the family is the closest circle that plays an important role in maintaining and helping the recovery process of people who experience psychosocial disorders. Family care and support will accelerate their recovery process. Family psychoeducation is part of psychosocial therapy, with the aim that families know about mental disorders and can reduce the relapse rate of PWMD. Strengthening family capacity can be influenced by support or assistance from health cadres who offer support via community mental health services. Health cadres who manage community mental health provide psychoeducation (40, 41), an activity that is important in improving the ability of health cadres to help the community overcome problems. In addition, this is an approach for PWMD families to proactively consult about the condition of one of their members.

Field visits by health volunteers to conduct family assessments of people with mental disabilities are important, but they are not always implemented as expected. There are various problems in conducting site visits, and more support is needed to enable health volunteers to fulfill their role. Technological advances, namely telenursing as part of telehealth, offer collaborative programs and reduce patient costs. Families can consult with health workers on a massive scale using this technology. Creating innovations as soon as possible to provide support to families with PWMD is essential; one technique could be to form groups of PWMD caregivers.

We need to focus not only on the person with mental illness, but also on the family as a whole in caring for their family member with mental illness (42, 43). The more challenging behaviors exhibited by the diagnosed person are associated with higher family discord. The Mental Illness Scale shows some capacity to measure the distress associated with having a family member with a mental illness (44). Results showed an improvement for family caregivers of the same size, indicating basic social functioning, less impairment in activities, and reduced feelings of guilt. Results also showed a meaningful decrease in the complexity of coping with unwanted negative indications of an ill family member and an increase in beneficial events and relationships with the family member (44, 45). Health care services should include group and family psychoeducation interventions as they have an excellent opportunity to reduce family burden (40). Inexhaustible psychoeducational family support groups contribute prolonged relief and facilitate troubled families who neglect family therapy.

## Conclusion

The research findings indicate that families resort to confining family members with mental disabilities due to a lack of understanding about their care and concerns about the impact of their behavior on the family and environment. The decision to confine is driven by the family's desire for comfort, supervision, and prevention of the patient getting lost. Factors such as poverty and the stigma surrounding mental illness also influence this decision. The research emphasizes the importance of psychoeducation for families to enhance their coping abilities and support the recovery process. It suggests the involvement of health cadres, psychoeducational programs, field visits, and telenursing as means to provide ongoing support and consultation. Overall, there is a need for family-focused interventions and support to address the challenges faced by families and promote the wellbeing of individuals with mental disorders and their families.

Suggestions for future research include investigating knowledge gaps, exploring long-term effects, comparing intervention effectiveness, examining healthcare professionals' roles, and exploring the experiences of individuals with mental disabilities. Addressing these areas can improve understanding and support for families and individuals with mental disorders.

### Recommendation

The researcher suggests that confinement is used as a final solution for the family and discusses the specifications of confinement and the family's purpose for confinement.

### Limitations

Qualitative research that was conducted in depth and focused on certain subjects and areas in the Province of South Sumatera, Indonesia, became a limitation of the study because it was difficult to generalize the research results to a wider population.

## Data availability statement

The original contributions presented in the study are included in the article/supplementary material, further inquiries can be directed to the corresponding author.

## Ethics statement

The studies involving human participants were reviewed and approved by Health Research Ethics Committee of the Palembang Health Polytechnic with an ethics certificate number 1271/KEPK/Adm2/IX/2021 on September 8, 2021. The patients/participants provided their written informed consent to participate in this study. Written informed consent was obtained from the individual(s) for the publication of any potentially identifiable images or data included in this article.

## Author contributions

IK conceived the original idea, designed and directed the project, verified the analytical methods, and took the lead in writing the manuscript. YY contributed to sample preparation, processed the data, and performed the analysis.
